# Circulating Levels of Cathelicidin Antimicrobial Peptide (CAMP) Are Affected by Oral Lipid Ingestion

**DOI:** 10.3390/nu15133021

**Published:** 2023-07-03

**Authors:** Alexandra Höpfinger, Thomas Karrasch, Andreas Schäffler, Andreas Schmid

**Affiliations:** Department of Internal Medicine III, University of Giessen, Klinikstr. 33, 35392 Giessen, Germany; thomas.karrasch@innere.med.uni-giessen.de (T.K.); andreas.schaeffler@innere.med.uni-giessen.de (A.S.); andreas.schmid@innere.med.uni-giessen.de (A.S.)

**Keywords:** CAMP, cathelicidin, bile acid, lipid, adipocyte

## Abstract

Introduction: Obesity and related diseases are among the main public health issues in the western world. They are thought to be caused by a state of chronic, low-grade inflammation. Cathelicidin antimicrobial peptide (CAMP) was recently discovered to be expressed and secreted by adipocytes. Representing a novel immunomodulatory adipokine, CAMP might play an important role in the complex interaction between metabolism and inflammation. Methods: In a cohort of 80 volunteers, serum samples were collected prior to, and 2 h, 4 h, and 6 h after, oral lipid ingestion. CAMP, fatty acid binding proteins 2 and 4 (FABP-2/-4), and dipeptidylpeptidase-4 (DPP-4) serum concentrations were measured via ELISA. Human Simpson–Golabi–Behmel syndrome (SGBS) adipocytes were treated with free fatty acids, and gene expression levels of *CAMP*, *FABP-4*, and *DPP-4* were quantified by RT-PCR. Results: The mean base-line CAMP serum concentration was 55.78 ± 29.26 ng/mL, with a range of 10.77–146.24 ng/mL. Interestingly, CAMP serum levels were positively correlated with LDL cholesterol, but negatively correlated with HDL cholesterol and adiponectin. Men exhibited higher CAMP serum concentrations than women, an effect apparently linked to oral contraception in the majority of female participants. In both genders, CAMP serum concentrations significantly decreased in a stepwise manner 4 h and 6 h after oral lipid ingestion. This decline was paralleled by a rise of serum bile acid and triglyceride levels upon lipid ingestion. In human SGBS adipocytes, treatment with free fatty acids did not affect *CAMP* gene expression, but increased *FABP-4* gene expression. Conclusions: In conclusion, systemic levels of the antimicrobial peptide and novel adipokine CAMP are significantly decreased upon oral lipid ingestion. While this decline might be linked to the simultaneous increase in bile acids, the underlying mechanisms remain to be elucidated. Furthermore, CAMP might indicate a putative novel cardiovascular biomarker of both inflammatory and metabolic relevance in metaflammation and adipose inflammation.

## 1. Introduction

Obesity and metabolic syndrome (MS) are among the main health risks worldwide [1]. The incidence of obesity increases progressively, not only in adults but also in children and adolescents [1]. Alongside this development, the prevalence of obesity-associated diseases, such as diabetes mellitus type 2 (T2D), hypertension, cardiovascular diseases (CVD), and dyslipidaemia, is increasing significantly [2]. Therefore, the prevention and treatment of obesity and obesity-related diseases are of great importance. However, due to the complexity of the endocrine organ adipose tissue, numerous underlying mechanisms remain yet to be elucidated.

Adipose tissue consists of different cell types, like adipocytes and stromal vascular cells (e.g., macrophages, lymphocytes, endothelial cells and stromal cells) [3]. During the development of obesity, the composition of visceral adipose tissue is substantially changed [4]. Polarization of anti-inflammatory M2-polarized macrophages changes to pro-inflammatory M1-polarized macrophages [5], and quantities of adipose tissue resident macrophages increase dramatically in obese individuals [4]. Furthermore, CD4+ T-cells [6] and natural killer cells infiltrate adipose tissue [7]. Accompanying these changes in adipose tissue composition, hypertrophic adipocytes in visceral obesity exhibit a modulated pattern of adipokine secretion [4]. In lean individuals, adipocytes express a balanced set of pro- and anti-inflammatory adipokines [8]. In obese individuals, proinflammatory adipokines, like monocyte chemotactic protein-1 (MCP-1), interleukin-6 and resistin, are predominantly secreted [9]. This increasingly proinflammatory environment induces a local and, in consequence, also systemic state of chronic low-grade inflammation [4]. This so-called metaflammation is thought to result in obesity-related diseases, summarized in the term MS [4]. Of note, secreted adipokines have immune-modulating abilities [4,10]. Adipokines, like leptin, modulate cytokine response in inflammatory settings and activate immune cells [11]. In obesity, dysregulated adipokine secretion thus perpetuates systemic low-grade inflammation [4].

Cathelicidin antimicrobial peptide (CAMP) represents a component of the innate immune system, being predominantly secreted by immune cells, like macrophages, natural killer cells, and lymphocytes [12]. In 2015, Zhang et al. reported that CAMP is produced by adipocytes of subcutaneous adipose tissue [13]. Therefore, CAMP can be considered as a novel adipokine. Functional adipogenesis in subcutaneous adipose tissue is crucial for local host defense [13]. Adipocytes and adipose tissue oppose Gram-positive bacterial infection of the skin by releasing CAMP [13], with the new term “killer fat” highlighting this significant importance of adipose tissue in host defense [14]. In murine adipocytes, *CAMP* expression is increased by enhanced Toll-like receptor (TLR)2/TLR4 activity [15]. Furthermore, *CAMP* gene expression in murine adipocytes is significantly reduced by metabolic factors, such as glucose and insulin, as well as incretins [16]. Bile acids, like tauromuricholic acid or taurohyodeoxycholic acid, increase *CAMP* gene expression in murine adipocytes [16]. However, overall data on the regulation and function of CAMP in human adipocytes is sparse so far. In severely obese patients, CAMP gene expression was found to be significantly higher in subcutaneous than visceral adipose tissue [16]. Of note, systemic levels of CAMP were observed to increase during weight loss induced by bariatric surgery, but not during weight loss by a low calorie formula diet [16]. In our previously published obesity cohort, a gender-related difference in CAMP serum levels was observed [16]. The underlying mechanisms remain unknown so far. Hormonal distinction might be causative for this effect.

Insights into the metabolic regulation of CAMP, both as a novel adipokine and a component of the innate immune system, might substantially improve our understanding of the complex interrelation of metabolism and immunity in obesity, metaflammation, and related comorbidities. Most importantly, the knowledge of underlying molecular mechanisms might provide new promising approaches for future therapeutic targets in the treatments of these disease entities.

Thus, the aim of the present study was to identify and characterize correlations of systemic CAMP with anthropometric, physiological, and metabolic parameters in human individuals without a history of diabetes mellitus, cardiovascular diseases or any other known preexistent illness. Furthermore, we investigated the short-term effects of oral lipid ingestion on systemic CAMP alongside FABP-2, FABP-4, and DPP-4 levels. Additionally, we aimed to elucidate the effect of free fatty acids on *CAMP* expression in human adipocytes in vitro.

## 2. Materials and Methods

### 2.1. SGBS Adipocyte Cell Culture and Stimulation Experiments

The Simpson–Golabi–Behmel syndrome (SGBS) preadipocyte cell strain are primary human cells that originated from adipose tissue specimens of a patient suffering from SGBS [17]. SGBS adipocytes are characterized by a high capacity for in vitro differentiation and, therefore, adequate for studies on mature human adipocytes. Cells were kindly provided by Prof. Martin Wabitsch (University of Ulm, Germany). Differentiation into mature adipocytes within 14 days was performed following the provider’s established protocol [17]. SGBS preadipocytes were cultured in DMEM/F12 (1:1) (Invitrogen, Darmstadt, Germany). DMEM/F12 (1:1) was supplemented with 10% FCS (purchased from Sigma-Aldrich, Deisenhofen, Germany). At 70–80% confluence, differentiation into mature adipocytes was induced. After a washing step with phosphate-buffered saline (PBS), cells were cultured in serum-free medium supplemented with 0.01 g/mL transferrin, 20 nM insulin, 0.2 nM triiodothyronine, and 100 nM cortisol (all purchased from Sigma-Aldrich, Deisenhofen, Germany). A total of 250 μM isobutylmethylxanthine (IBMX), 2 μM rosiglitazone (BRL 49653) (Cayman, Tallinn, Estonia), and 25 nM dexamethasone (all purchased from Sigma-Aldrich, Deisenhofen, Germany) were added to the medium during the first 4 days of differentiation. Every four days, the culture medium was replaced. The characteristic morphology of preadipocyte stages and of mature adipocytes was visually controlled by light microscopy. Mature adipocytes were incubated under serum-free conditions prior to stimulation experiments. Selected doses of stearic acid (200 µM), palmitoleic acid (50 µM) and docosahexaenoic acid (50 µM) were applied during overnight (18 h) stimulation experiments. The applied stimulatory doses had been determined either by preliminary tests or previous experiments in adipocyte culture with respect to dose effects and cell viability [18]. Furthermore, LDH (lactate dehydrogenase) activity was measured in the cell supernatants (Cytotoxicity Detection Kit, Roche, Mannheim, Germany) of all experimental samples, in order to exclude any unintended cytotoxic effects.

### 2.2. Gene Expression Analysis in SGBS Adipocytes

Reverse transcription of RNA (QuantiTect Reverse Transcription Kit from Qiagen, Hilden, Germany) was performed in order to generate corresponding cDNA, followed by a quantitative real-time PCR (RT-PCR) (iTaq Universal SYBR Green Supermix, CFX Connect RT-PCR system; Bio-Rad, Munich, Germany). *CAMP*, *DPP4*, and *FABP4* gene expression was normalized to the gene expression of glyceraldehyde-3-phosphate dehydrogenase (*GAPDH*) as a house-keeping gene, applying the ΔΔCT method. The following primer-pairs were applied:

Human *CAMP*:
5′-TAGATGGCATCAACCAGCGG-3′/5′-CTGGGTCCCCATCCATCGT -3′
Human *DPP4*:
5′-TTCTGCTGAACAAAGGCAATGA-3′/5′-CTGTTCTCCAAGAAAACTGAGCTG-3′
Human *FABP4*:
5′-ATGGGGGTGTCCTGGTACAT-3′/5′-CTTTCATGACGCATTCCACCA-3′
Human *GAPDH*:
5′-GAGTCCACTGGCGTCTTCAC-3′/5′- CCAGGGGTGCTAAGCAGTT-3′
All oligonucleotides used were purchased from Metabion, Martinsried, Germany.

### 2.3. Study Cohort

A total of 80 (50 females and 30 males) adult volunteers participated in this study at the University Hospital Regensburg, Germany. The study was approved by the local ethical committee (ethical committee of the University Hospital Regensburg, 11-101-0058, date of approval: 31 March 2011) and all individuals participating gave informed consent.

The impact of oral lipid tolerance test (OLTT) was investigated. A lipid solution containing all types of fatty acid species (saturated, mono-unsaturated and poly-unsaturated fatty acids), and free of proteins and carbohydrates, was applied. A total of 160 mL of a preparation from 3 different lipid solutions were applied, containing 758.1 kcal, with 75 g vegetable fat as triglycerides and 9.2 g fatty acids as pure vegetable oils. After overnight fasting, blood samples were taken at 0 h (prior to start of the trial), 2 h, 4 h, and 6 h after oral lipid ingestion. Patients with a history of diabetes mellitus, cardiovascular diseases or any kind of other known preexistent illness were excluded from the study. Individuals with evidence of acute or chronic infection within the last 10 days, pregnancy, age < 18 years or >55 years, and any kind of medication except oral contraceptives were not admitted to the study. More detailed information on the composition of the lipid solution, study protocol and basic anthropometric data (e.g., age, BMI, hip circumference, waist circumference, waist/hip ratio, triceps skinfold thickness, blood pressure, patients’ history concerning type 2 diabetes and cardiovascular diseases, as well as habits such as smoking and hormonal contraception) was previously described in detail [19]. Measurement of skinfold thickness was performed at the dorsal side of musculus triceps brachii with a skinfold caliper.

### 2.4. Measurement of Systemic Adipokine Concentrations

Levels of serum CAMP were quantified in duplicates by ELISA (Hycultec, Beutelsbach, Germany) and are expressed as means ± standard deviation. The lower detection limit was 0.14 ng/mL. Levels of FABP-2, FABP-4 and DPP-4 were quantified in duplicates by ELISA (DuoSet ELISA Development Kits, Bio-Techne, Wiesbaden, Germany), and are expressed as means ± standard deviation (lower detection limit FABP-2: 31.2 pg/mL; lower detection limit FABP-4: 62.5 pg/mL; lower detection limit DPP-4: 31.2 pg/mL).

Furthermore, serum concentrations of adipokines like adiponectin and resistin were measured by ELISA and published previously [20]. The present study cohort represents a sub-cohort of our initial study [19], and was analyzed for serum CAMP, FABP-2, FABP-4 and DPP-4 levels for the first time.

### 2.5. Measurement of Metabolic and Anthropometric Parameters

At the Institute of Clinical Chemistry and Laboratory Medicine at the University of Regensburg, Germany, the measurement of plasma glucose, plasma insulin, C-reactive protein (CRP), total cholesterol, triglycerides, high-density lipoprotein cholesterol (HDL), and low-density lipoprotein cholesterol (LDL) was performed, applying standard techniques (Table 1). Bile acids (total bile acids; subgroups of free bile acids, taurine-conjugated bile acids, and glycine-conjugated bile acids) were quantified by liquid chromatography–tandem mass spectrometry and published earlier [21]. In a previously published study, routine parameters and characteristics of the study have been shown and discussed [19].

### 2.6. Statistical Analysis

**Figure 1:**
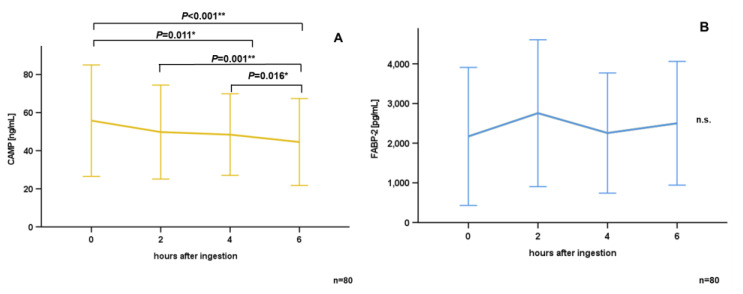

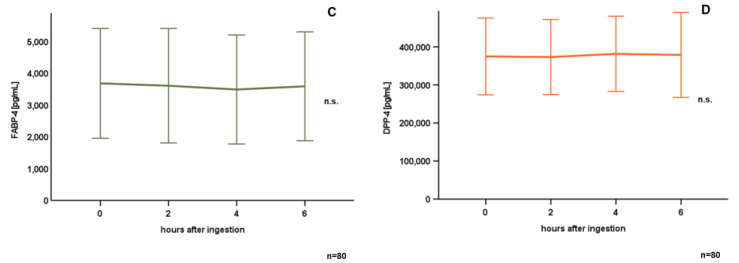
CAMP serum concentration significantly decreases during an oral lipid tolerance test in a stepwise manner. A total of 80 volunteers without a history of diabetes mellitus, cardiovascular diseases, or any kind of other known preexistent illness, ingested a lipid solution. CAMP (**A**), FABP-2 (**B**), FABP-4 (**C**) and DPP-4 (**D**) serum concentrations were measured prior to, and 2 h, 4 h, and 6 h after, oral lipid ingestion via ELISA. The Wilcoxon test was applied for the calculation of statistical significance (* *p* < 0.05, ** *p* < 0.01). Results are presented as mean ± SD, n = 80. n.s.: not significant.

**Figure 2:**
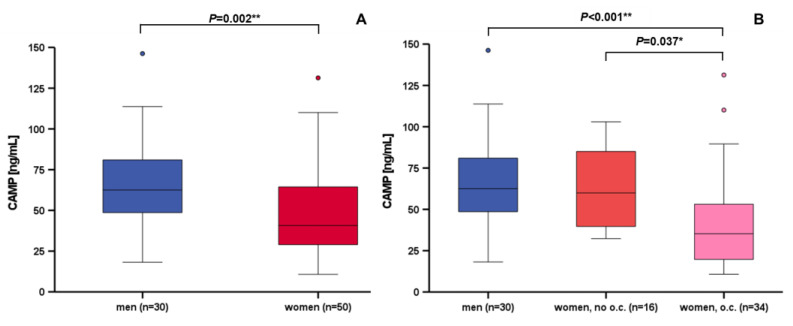
The sexual dimorphism of CAMP concentrations is caused by oral contraception. CAMP serum concentrations were measured in 80 volunteers. Men (n = 30) exhibit higher CAMP serum levels than women (n = 50, with and without oral contraception) (mean ± SD: men: 67.29 ng/mL ± 27.42; women: 48.88 ng/mL ± 28.39) (**A**). Women without oral contraception (n = 16) have higher CAMP serum levels than women taking oral contraceptives (n = 34), equaling men’s CAMP concentrations (**B**). CAMP serum concentrations were quantified by ELISA. The Mann–Whitney U test was applied for calculation of statistical significance (* *p* < 0.05, ** *p* < 0.01), and the data are presented as box plots. o.c. oral contraception. mean ± SD: men: 67.29 ng/mL ± 27.42; women no o.c.: 61.92 ng/mL ± 24.94; women with o.c.: 42.75 ng/mL ± 28.16.

A statistical software package (SPSS 28.0.0.0) was used for the calculation of mean values ± standard deviation (±SD). CAMP adipocyte gene expression levels and serum concentrations did not follow a Gaussian distribution. Non-parametric numerical parameters were analyzed by the Mann-Whitney U test (for 2 unrelated samples), the Kruskal-Wallis test (>2 unrelated samples), or the Friedman test (>2 related samples). Correlation analysis was performed applying the Spearman-rho test for linear variables. *p* values below 0.05 (two tailed) were considered statistically significant. In Figure 1 and Figure 4, the results are visualized as mean ± standard deviation. In Figure 2 and Figure 5, the results are visualized as boxplots, giving the median and the quartile ranges. 

## 3. Results

### 3.1. Characteristics of the Study Cohort

A total of 80 volunteers (50 women and 30 men) were included in the present study. Patients with a history of diabetes mellitus, cardiovascular diseases, or any kind of other known preexistent illness were excluded from the study. Table 1 summarizes the characteristics of the study cohort. The age ranged between 18 and 54 years. A total of 28 individuals were overweight or obese, with a BMI ≥ 25kg/m², and 52 had normal weight, with a BMI < 25kg/m². Parameters of lipid and glucose metabolism, such as total cholesterol, triglycerides, HDL cholesterol, LDL cholesterol, glucose, and insulin, were recorded. Base-line serum concentrations of CAMP (mean 55.78 ± 29.26 ng/mL), FABP-2 (2171.59 ± 1739.56 pg/mL), FABP-4 (3686.09 ± 1728.77 pg/mL), and DPP-4 (374.93 ± 100.93 ng/mL) were quantified. A more detailed characteristic of the study cohort is shown in Supplementary Table S1.

### 3.2. Oral Lipid Ingestion Reduces CAMP but Does Not Affect FABP-2, FABP-4, and DPP-4 Serum Concentrations

Systemic CAMP levels were quantified in serum samples obtained immediately before, 2 h, 4 h, and 6 h after oral lipid ingestion. As demonstrated in Figure 1A, mean CAMP serum concentrations were reduced 4 h (*p* = 0.011*) and 6 h (*p* < 0.001**) after oral lipid ingestion when compared to base-line levels. Furthermore, CAMP serum concentrations were reduced in a stepwise manner from 2 h to 4 h (*p* = 0.001**), and from 4 h to 6 h (*p* = 0.016*). Furthermore, FABP-2 (Figure 1B), FABP-4 (Figure 1C), and DPP-4 (Figure 1D) serum concentrations were measured at the determined time-points. Unlike CAMP, there was no significant modulation of these proteins after oral lipid uptake.

### 3.3. Correlation and Subgroup Analysis of Systemic CAMP Levels

Subgroup analyses were performed by the Mann–Whitney U test. Men exhibited significantly higher CAMP serum concentrations than women (*p* = 0.002**) (Figure 2A). Of note, women applying oral contraception had significantly decreased CAMP levels when compared to men (*p* < 0.001**) or to women without oral contraception (*p* = 0.037*) (Figure 2B). Furthermore, the decline of CAMP serum concentrations after oral lipid ingestion was significantly higher in women without oral contraception (*p* = 0.008*) (Supplementary Figure S1). In BMI categories (category 1: <25 kg/m²; category 2: ≥25 kg/m²), there was neither a significant difference in base-line CAMP serum concentrations (Supplementary Figure S2A), nor in their dynamics after oral lipid ingestion (Supplementary Figure S2B).

**Figure 3:**
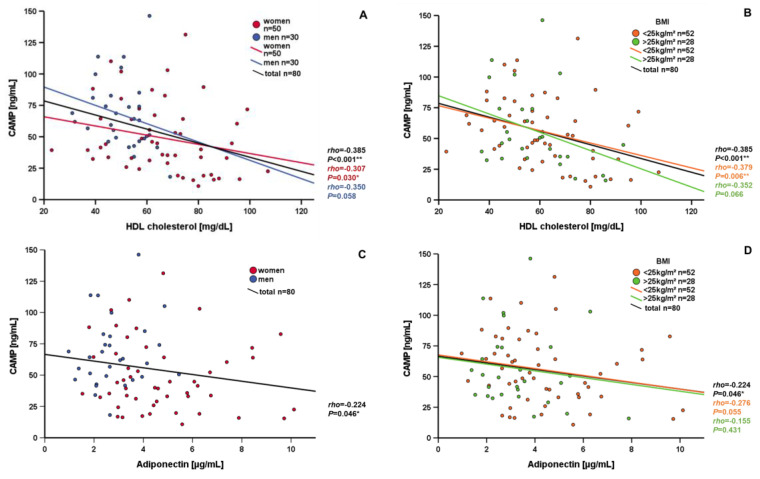

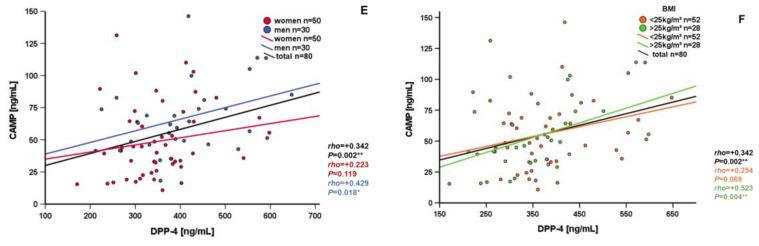
Negative correlation of CAMP serum concentrations with cardioprotective parameters, and positive correlation with DDP-4 serum levels in volunteers. Serum CAMP and HDL cholesterol levels are negatively correlated in the total study cohort of 80 volunteers without a history of diabetes mellitus, cardiovascular diseases or any kind of other known preexistent illness (**A**,**B**). This correlation is specific for the subgroups of women (**A**) and of normal weight individuals (**B**). Serum adiponectin and CAMP concentrations are negatively correlated (n = 80) (**C**,**D**). DPP-4 and CAMP levels are positively correlated, specifically in male (**E**) and overweight individuals (**F**). CAMP serum concentrations were measured by ELISA. Spearman-rho test was applied for calculation of correlation coefficient and statistical significance (* *p* < 0.05, ** *p* < 0.01).

Correlation analyses of CAMP with metabolic serum parameters with significant results are summarized in Table 2A, insignificant results are shown in Supplementary Table S5. CAMP serum concentrations were negatively correlated with skinfold thickness (*p* < 0.001**), but there was no relation to other morphological markers of obesity, like waist-to-hip ratio, body weight, or BMI. A significant negative correlation of CAMP and skinfold thickness persisted after the exclusion of possible confounders by partial correlation analyses, like age, BMI, triglycerides, LDL cholesterol, or total cholesterol (Supplementary Table S2). HDL cholesterol levels correlated negatively with CAMP serum concentrations in the entire study population (*p* < 0.001**) (Figure 3A,B), as well as in subgroups of women (*p* = 0.030*) (Figure 3A), in patients with an BMI < 25 kg/m² (*p* = 0.006*) (Figure 3B), and in non-smokers (*p* = 0.002*) (Supplementary Figure S3), but not in subgroups of patients with an BMI ≥ 25kg/m², men, or smokers. This significant negative correlation persisted after exclusion of possible confounders by partial correlation analyses like age, BMI, triglycerides, LDL cholesterol, or total cholesterol (Supplementary Table S3). In contrast to HDL, LDL cholesterol levels correlated positively with CAMP serum concentrations in a subgroup of normal-weight patients with a BMI of <25 kg/m² (*p* = 0.013*) (Table 2B), low WHR (*p* = 0.018*) (Supplementary Figure S4A), and small skinfold thickness (*p* = 0.007*) (Supplementary Figure S4B). CAMP concentration correlated positively with triglycerides (*p* = 0.020*) in patients with BMI < 25 kg/m² (Table 2B). In patients with BMI ≥ 25 kg/m², CAMP serum concentrations correlated negatively with skinfold thickness (*p* < 0.001**) and insulin (*p* = 0.049*) (Table 2B). Adiponectin and CAMP correlated negatively in the entire study population (Figure 3C). Significant confounders might be age and BMI (Supplementary Table S4). This correlation did not reach statistical significance in the subgroups of normal weight and obese patients (Figure 3D). Systemic DPP-4 concentrations correlated positively with CAMP serum levels in patients with BMI ≥ 25 kg/m² (*p* = 0.004**) (Figure 3F, Table 2B) and with WHR > 0.84 (*p* = 0.007*) (Supplementary Figure S5A), in men (*p* = 0.018*) (Figure 3E), and in non-smokers (*p* = 0.001**) (Supplementary Figure S5B). The decrease in CAMP serum concentrations 6 h after oral lipid ingestion (ΔCAMP) correlated negatively with basal DPP-4 concentration, whereas other basal metabolic parameters, such as BMI, skinfold thickness, triglycerides, cholesterol, glucose, or insulin, were not correlated (Table 2A). Changes in metabolic parameters after oral lipid ingestion (Δ metabolic parameters) were not associated with the decrease in CAMP serum concentrations (Table 2A).

**Figure 4:**
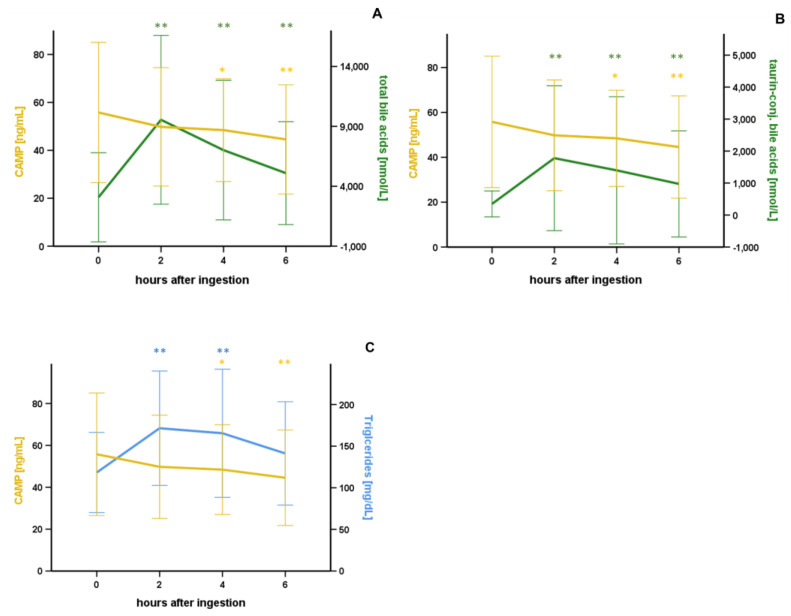
The significant decline in CAMP concentrations parallels the increase in systemic bile acid levels after oral lipid ingestion. CAMP serum concentrations decrease upon oral lipid ingestion, in contrast to a strong initial rise of total (**A**), taurine-conjugated bile acids (**B**), and triglyceride quantities (**C**). CAMP serum concentration was investigated by ELISA. Bile acids were quantified by liquid chromatography-tandem mass spectrometry earlier [21]. * *p* < 0.05, ** *p* < 0.01 compared to baseline. Results are presented as mean ± SD. n = 80.

With serum levels of bile acids having been described earlier [21], we now investigated a potential interrelation of bile acids and CAMP in serum after oral lipid ingestion for the first time. Importantly, basal CAMP concentrations correlated negatively with taurine-conjugated bile acids (*p* = 0.013*), but not with total, free, or glycine-conjugated bile acids (Table 3). Furthermore, the decrease in CAMP serum concentrations (ΔCAMP) within 6 h (*p* = 0.001**) and 2 h (*p* < 0.001**) was significantly correlated with basal taurine-conjugated bile acid levels (Table 3). In the subgroup of patients with BMI ≥ 25 kg/m², serum CAMP concentrations were negatively correlated with taurine- (*p* = 0.001**) and glycine-conjugated bile acid levels (*p* = 0.044*), but not in patients with BMI < 25 kg/m² (Table 3). Insignificant correlation analyses of CAMP and bile acids are shown in Supplementary Table S6. The decrease in CAMP concentrations was associated with, and paralleled by, a substantial initial increase in total bile acids (Figure 4A), taurine-conjugated bile acids (Figure 4B), and triglycerides (Figure 4C).

### 3.4. Effect of Free Fatty Acids on CAMP Expression in Human SGBS Adipocytes In Vitro

**Figure 5:**
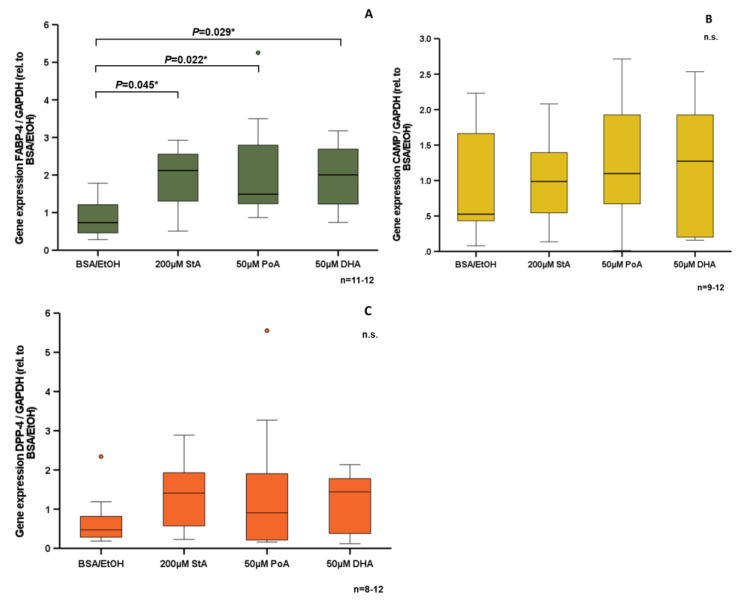
Free fatty acids induce *FABP-4* gene expression in SGBS adipocytes in vitro. SGBS adipocytes were stimulated with 200 µM StA (stearic acid), 50 µM PoA (polyamic acid), and 50 µM DHA (docosahexaenoic acid) in vitro. Compared to solvent control BSA/EtOH (bovine serum albumin/ethanol), *FABP-4* mRNA levels were increased by stimulation with free fatty acids (**A**). *CAMP* (**B**) and *DPP-4* (**C**) gene expression were not affected by free fatty acids. Gene expression levels were analyzed by quantitative real-time PCR. The Kruskal–Wallis test was applied for calculation of statistical significance (* *p* < 0.05). N = 8–12 wells were investigated per experimental setting. n.s.: not significant.

Having observed significantly reduced CAMP serum concentrations upon lipid ingestion in vivo, we investigated the effect of free fatty acids on human SGBS adipocytes in vitro. Stearic acid (*p* = 0.045*), palmitoleic acid (*p* = 0.022*), and docosahexaenoic acid (*p* = 0.029*) each increased *FABP-4* expression significantly (Figure 5A). In contrast, these free fatty acids had neither a significant effect on *CAMP* (Figure 5B) nor on *DPP-4* expression in vitro (Figure 5C).

## 4. Discussion

As a novel adipokine, the antimicrobial peptide CAMP might play an important role in the context of metaflammation and adipose inflammation.

Data on CAMP serum concentrations in healthy volunteers is sparse because most studies involving patient cohorts investigated disease-related questions. In our cohort of subjects without a history of diabetes mellitus, cardiovascular diseases, or any kind of other known preexistent illness, the mean CAMP serum concentration was 55.78 ± 29.26 ng/mL, slightly higher than in morbidly obese patients [16]. Nonetheless, CAMP serum concentrations were not correlated with BMI in our participants. Due to the high number of participants (n = 80) undergoing an oral lipid tolerance test, we consider the presented data on basal and postprandial systemic CAMP to be of high scientific relevance. In obesity, a state of low-grade inflammation is observed. Visceral adipose tissue is infiltrated by immune cells, and proinflammatory adipokines and cytokines are released systematically [4]. The role of CAMP serum levels in this context is not fully understood so far. Therefore, proinflammatory adipokines and cytokines might downregulate CAMP serum levels.

Since obesity is associated with cardiovascular diseases [22], we investigated cardiovascular risk factors. LDL cholesterol levels correlated positively with CAMP serum concentrations in patients with normal weight (BMI < 25 kg/m²), low WHR, and a low skinfold thickness. Skinfold thickness is thought to be a predictor of the total quantity of body fat and, more specifically, of subcutaneous adipose tissue. The persistent positive correlation of LDL cholesterol and CAMP after exclusion of potential confounders indicates a relevant quantity of CAMP originated from adipose tissue. Furthermore, HDL cholesterol correlated negatively with systemic CAMP in the total study population, even after exclusion of possible confounders by partial correlation analysis. As another cardioprotective parameter, serum adiponectin was negatively correlated with CAMP. Due to the negative correlation of CAMP serum concentrations with cardioprotective parameters, and positive correlation with the cardiovascular risk factor LDL cholesterol, CAMP might represent a novel potential biomarker for metabolic risk. In mice, CAMP was found to act as a potential self-antigen involved in immune response to atherosclerosis [23]. Taken together with our findings, CAMP might play a role in human atherosclerosis.

We further observed that female individuals exhibited significantly lower CAMP serum concentrations than males. Similar findings have been reported in other cohorts [16,24]. In our present study, this sexual dimorphism was substantially based on the application of oral contraception. This observation is new, and has not been reported so far. In vitro, gender-specific hormones do not modify CAMP gene expression in adipocytes, as was recently reported [16]. Therefore, adipocytes might not represent the primary site of gender-dependent regulation of CAMP gene expression. However, future studies will be needed to adequately address this intriguing issue in further detail and on a mechanistic level.

Metabolic health and adipokine secretion strongly depend on diet and physical activity [25]. Regular physical activity improves the metabolic state and enhances insulin sensitivity [26], whereas diets predominantly consisting of simple carbohydrates or saturated fatty acids favor the development of insulin resistance [27]. Free fatty acids are translocated via fatty acid binding proteins (FABP) into the cellular compartments [28]. FABPs play a significant role in regulating metabolism, and are linked to the development of insulin resistance and the metabolic syndrome [29]. Oral lipid and glucose intake modulate adipokine concentrations in serum [19,20,30]. Our present study investigated, for the first time, the short-term regulation of CAMP serum concentrations during a highly defined oral lipid tolerance test. The used carbohydrate- and protein-free lipid solution contained a defined composition of lipids. CAMP serum concentrations significantly decreased upon lipid ingestion in a stepwise manner. This important physiological regulation is new, and has not yet been described in the literature. Whether specific free fatty acids or triglycerides, or the composition of the lipid solution, caused the decrease of CAMP serum concentration remains to be elucidated. Furthermore, a mixed meal from a Western diet or isolated carbohydrate ingestion might have divergent effects on postprandial CAMP serum concentrations. Nonetheless, together with CAMP’s well-established role as an antimicrobial peptide, this finding suggests that it might represent a relevant factor in the immune-metabolic crosstalk, although the underlying mechanisms remain to be elucidated. In contrast to the observed dynamics of CAMP concentrations, serum levels of metabolic parameters, such as FABP-2, FABP-4, or DPP-4, were not altered by lipid intake, unlike various adipokines, such as visfatin [19].

The significant and sustained decrease in CAMP parallels with a strong initial increase in bile acids after oral lipid ingestion [21]. Bile acids are able to modify the secretion of adipokines in adipocytes in vitro, such as adiponectin and resistin [31]. Bile acid concentrations rise 2 h after oral lipid ingestion, as previously reported by our group [21]. Basal concentrations of taurine-conjugated bile acids were negatively correlated with basal CAMP concentration, as well as with the decrease in CAMP serum concentration 2 and 6 h after oral lipid ingestion. We previously demonstrated that taurine-conjugated bile acids, α-tauromuricholic acid (TMCA), and taurohyodeoxycholic acid (THDCA) significantly induce CAMP gene expression in adipocytes in vitro, whereas taurodeoxycholic acid (TDCA) does not affect CAMP gene expression [16]. Therefore, a potential modulation of CAMP gene expression during the early increase in taurine-conjugated bile acid concentrations after oral lipid ingestion might be specific to certain bile acid species. In biliary epithelial cells, regulation of CAMP expression by bile acids via two different nuclear receptors has been reported earlier [32]. It, therefore, appears reasonable to assume that the observed decrease in CAMP serum concentrations during OLTT may be directly linked to an increase in bile acids; however, identification of the respective underlying mechanism requires further investigation.

Systemic triglycerides exhibited similar dynamics to those of bile acids upon lipid ingestion [19]. In SGBS adipocytes, free fatty acids did not modulate in vitro *CAMP* gene expression. Therefore, the increase of triglycerides might not be causative for *CAMP* reduction during an oral lipid tolerance test, and data from in vitro experiments do not support a role of adipocytes as a celltype responding to fatty acids as nutritive CAMP regulators. At least, our data indicated no relevant influence of the tested free fatty acids on CAMP gene expression in human adipocytes. Nonetheless, other free fatty acids might influence CAMP gene expression in human adipocytes. Additionally, studies on protein levels are necessary to fully answer this question. Overall, it cannot be excluded that oral lipid uptake initiates mechanisms which affect adipocytic CAMP gene expression and secretion. Of note, CAMP is secreted by various cell types, like immune cells, adipocytes, and epithelial cells in human subjects [12], therefore, CAMP serum concentration probably does not only originate from adipocytes. Nonetheless, adipocytes and adipokines play an important role in metaflammation, thus elucidating CAMP regulation in adipocytes is of high relevance.

## 5. Conclusions

In conclusion, the present study investigated the antimicrobial peptide and novel adipokine CAMP in a large cohort of volunteers without a history of diabetes mellitus, cardiovascular diseases, or any kind of other known preexistent illness, undergoing an oral lipid tolerance test. Our data suggest that CAMP serum concentrations might represent a potential novel metabolic biomarker. Furthermore, CAMP might indicate a putative novel cardiovascular biomarker. Importantly, we demonstrate that CAMP serum concentrations significantly decrease in a stepwise manner upon oral lipid ingestion. Therefore, CAMP might play an important role at the interface of metabolism and immunity, especially in the onset and development of metaflammation. The decline of CAMP serum concentrations might also be physiologically linked to a strong increase in circulating bile acid levels. Such an interrelation might specifically involve taurine-conjugated bile acid species, with underlying mechanisms remaining to be elucidated.

## Figures and Tables

**Table 1:** Study cohort.

	Study Cohort (t = 0)
Gender (men/women)	30/50
Age [y]	28.30 ± 7.53 (18–54)
BMI [kg/m²]	24.25 ± 5.06 (14.80–46.10)
WHR	0.84 ± 0.09 (0.69–1.07)
HOMA Index	1.72 ± 1.43 (0.18-8.47)
Glucose [mg/dL]	74.49 ± 13.73 (19–110)
Total cholesterol [mg/dL]	190.56 ± 38.01 (52–297)
Triglycerides [mg/dL]	118.38 ± 48.17 (24–299)
HDL cholesterol [mg/dL]	60.64 ± 17.45 (23–107)
LDL cholesterol [mg/dL]	110.20 ± 31.33 (26–196)
CAMP [ng/mL]	55.78 ± 29.26 (10.77–146.24)

Basic characteristics of the study cohort are presented with mean ± standard deviation and (range). The investigated 80 volunteers are part of a larger cohort published earlier [19]. t = 0, base-line characteristics (prior to oral lipid ingestion).

**Table 2:** Correlation analysis of CAMP and metabolic parameters.

**A**		
**Metabolic Parameters (t = 0), n = 80**	**CAMP [ng/mL] (t = 0)**	
	*rho*	*p*
Skinfold [mm]	−0.384	<0.001 **
HDL cholesterol [mg/dL]	−0.385	<0.001 **
DPP-4 [pg/mL]	+0.342	0.002 **
**Metabolic Parameters (t = 0), n = 80**	**Δ** **CAMP [ng/mL] (t = (t = 6)–(t = 0))**	
DPP-4 [pg/mL]	−0.380	<0.001 **
**B**		
**Metabolic Parameters BMI < 25kg/m² (t = 0), n = 52**	**CAMP [ng/mL] (t = 0)**	
HDL cholesterol [mg/dL]	−0.379	0.006 **
LDL cholesterol [mg/dL]	+0.342	0.013 *
Triglycerides [mg/dL]	+0.321	0.020 *
**Metabolic Parameters BMI > 25kg/m² (t = 0), n = 28**	**CAMP [ng/mL] (t = 0)**	
Skinfold [mm]	−0.667	<0.001 **
Insulin [mU/L]	−0.375	0.049 *
DPP-4 [pg/mL]	+0.523	0.004 **

CAMP serum concentrations were measured by ELISA. Spearman-rho test was applied for calculation of correlation coefficient and statistical significance (* *p* < 0.05, ** *p* < 0.01). t = hours after oral lipid ingestion. (A) total study cohort (n = 80); (B) subgroup analyses: BMI < 25 kg/m² (n = 52); BMI ≥ 25 kg/m² (n = 28) (insignificant results are shown in Supplementary Table S5).

**Table 3:** Correlation analysis of CAMP and bile acids.

**Bile acids (t = 0), n = 80**	**CAMP [ng/mL] (t = 0)**	
	***rho***	***p***
Taurine-conj. Bile acids [nmol/L]	−0.276	0.013 *
**bile acids (t = 0), n = 80**	**Δ** **CAMP [ng/mL] (t = t6 – t0)**	
Taurine-conj. Bile acids [nmol/L]	+0.358	0.001 **
**bile acids (t = 0), n = 80**	**Δ** **CAMP [ng/mL] (t = t2 – t0)**	
Taurine-conj. Bile acids [nmol/L]	+0.422	<0.001 **
**bile acids, BMI > 25 kg/m² (t = 0), n = 28**	**CAMP [ng/mL] (t = 0)**	
Taurine-conj. Bile acids [nmol/L]	−0.572	0.001 **
Glycine-conj. Bile acids [nmol/L]	−0.383	0.044 *

CAMP serum concentrations were measured by ELISA. A Spearman-rho test was applied for calculation of the correlation coefficient and statistical significance. (* *p* < 0.05, ** *p* < 0.01). t = hours after oral lipid ingestion. (Insignificant results are shown in Supplementary Table S6).

## Data Availability

The data presented in this study are available on reasonable request from the corresponding author.

## Supplementary Materials

The following supporting information can be downloaded at: https://www.mdpi.com/article/10.3390/nu15133021/s1, Figure S1: The decline of serum CAMP levels after oral lipid ingestion was significantly higher in women without oral contraception; Figure S2: Comparing BMI categories (normal weight vs. overweight/obese), there was neither a significant difference in base-line CAMP serum concentrations (2A) nor in their dynamics after oral lipid ingestion (2B); Figure S3: HDL cholesterol levels correlated negatively with CAMP serum concentrations in the subgroup of non-smokers; Figure S4: LDL cholesterol levels correlated positively with CAMP serum concentrations in the subgroup of patients with low waist-hip-ratio (A) and small skinfold (B); Figure S5: DPP-4 levels correlated positively with CAMP serum concentrations in the subgroup of patients with elevated waist-hip-ratio (A) and non-smokers (B); Table S1: Study cohort; Table S2: Significant and positive correlation of CAMP and skinfold persisted after exclusion of confounders; Table S3: Significantly and negatively correlation of CAMP and HDL cholesterol persisted after exclusion of confounders; Table S4: Partial correlation analyses revealed age or BMI as confounders of significant negative correlation of CAMP and adiponectin; Table S5 Correlation analysis of CAMP and metabolic parameters; Table S6: Correlation analysis of CAMP and bile acids.
