# Supplementary material for: Circulating Levels of Cathelicidin Antimicrobial Peptide (CAMP) Are Affected by Oral Lipid Ingestion
Source: Nutrients. 2023 Jul 3;15(13):3021. doi: 10.3390/nu15133021 (PMC10347133; doi:10.3390/nu15133021)
Supplement: Supplementary file 1 [file nutrients-15-03021-s001.zip › nutrients-2445130-supplementary.pdf]

**A) men (n=30)**	**Study cohort (t = 0)**
Age [y]	27.47±7.59 [18-54]
BMI [kg/m²]	25.11±3.43 [19.9-34.6]
WHR	0.88±0.09 [0.75-1.07]
HOMA Index	1.51±0.93 [0.42-3.78]
Glucose [mg/dL]	73-73±12.01 [44-92]
Total cholesterol [mg/dL]	184.00±40.52 [111-297]
Triglycerides [mg/dL]	122.80±47.62 [51-254]
HDL cholesterol [mg/dL]	50.60±9.33 [31-69]
LDL cholesterol [mg/dL]	113.93±33.54 [51-196]
CAMP [ng/mL]	67.29±27.42 [18.18-146.24]
**B) women (n=50)**	**Study cohort (t = 0)**
Age [y]	28.80±7.53 [21-49]
BMI [kg/m²]	23.73±5.79 [14.8-46.1]
WHR	0.81±0.08 [0.69-0.98]
HOMA Index	1.84±1.65 [0.18-8.47]
Glucose [mg/dL]	74.94±14.72 [19-110]
Total cholesterol [mg/dL]	194.50±36.27 [52-277]
Triglycerides [mg/dL]	115.72±48.46 [24-299]
HDL cholesterol [mg/dL]	66.66±18.46 [23-107]
LDL cholesterol [mg/dL]	107.96±30.06 [26-174]
CAMP [ng/mL]	48.88±28.39 [10.77-131.33]
**C) normal weight (n=52)**	**Study cohort (t = 0)**
Gender (men/women)	15/37
Age [y]	26.87±6.50 [18-49]
BMI [kg/m²]	21.61±2.19 [14.8-24.9]
WHR	0.80±0.07 [0.69-0.93]
HOMA Index	1.32±0.71 [0.18-3.95]
Glucose [mg/dL]	73.65±12.70 [19-93]
Total cholesterol [mg/dL]	180.75±33.69 [52-255]
Triglycerides [mg/dL]	106.71±37.43 [24-184]
HDL cholesterol [mg/dL]	61.96±18.77 [23-107]
LDL cholesterol [mg/dL]	99.79±27.07 [26-147]
CAMP [ng/mL]	55.45±28.12 [10.77-131.33]
**D) overweight (n=28)**	**Study cohort (t = 0)**
Gender (men/women)	15/13
Age [y]	30.96±8.66 [22-54]
BMI [kg/m²]	29.15±5.24 [25.0-46.1]
WHR	0.90±0.08 [0.74-1.07]
HOMA Index	2.45±2.03 [0.56-8.47]
Glucose [mg/dL]	76.04±15.59 [45-110]
Total cholesterol [mg/dL]	208.79±39.42 [145-297]
Triglycerides [mg/dL]	140.04±58.28 [57-299]
HDL cholesterol [mg/dL]	58.18±14.70 [37-93]
LDL cholesterol [mg/dL]	129.54±29.86 [72-196]
CAMP [ng/mL]	56.41±31.81 [15.87-146.24]

Supplementary Table S1: **Study cohort**

Basic characteristics of the study cohort are presented with mean ± standard deviation and [range]. Patients were divided in subgroups: A) men; B) women; C) BMI < 25kg/m²; D) BMI ≥ 25kg/m²

The investigated 80 volunteers are part of a larger cohort published earlier [19]. t = 0, base-line characteristics (prior to oral lipid ingestion)

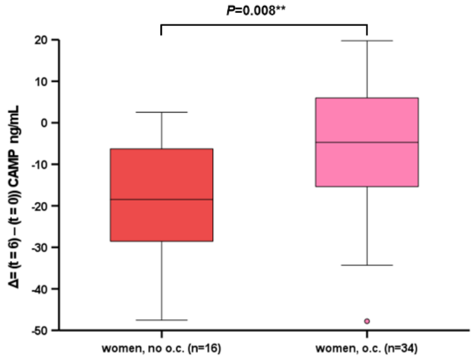

Supplementary Figure S1: **The decline of serum CAMP levels after oral lipid ingestion was significantly higher in women without oral contraception.**
The decline of CAMP serum levels (ΔCAMP = (t = 6) – (t = 0)) was determined for the subgroup of women. The decline of CAMP serum levels in women without oral contraception (n = 16) is higher compared to CAMP serum levels than women taking oral contraceptives (n = 34). CAMP serum concentrations were quantified by ELISA. The Mann-Whitney U-test was applied for calculation of statistical significance (** $p < 0.01$). o.c. oral contraception

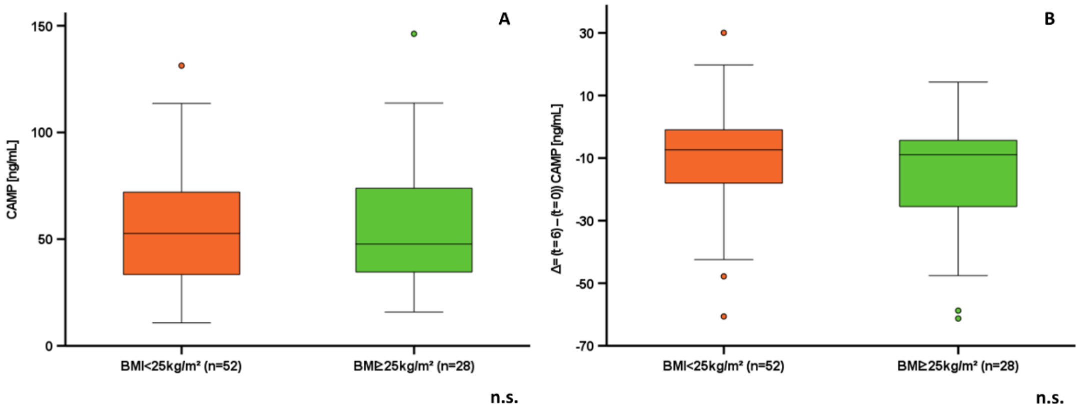

Supplementary Figure S2: Comparing BMI categories (normal weight vs. overweight/obese), there was neither a significant difference in base-line CAMP serum concentrations (2A) nor in their dynamics after oral lipid ingestion (2B)
Basal CAMP serum levels (2A) and the decline of CAMP serum levels (ΔCAMP) (2B) was measured in the subgroups of normal weight and overweight patients. There is no significant difference between these subgroups. CAMP serum concentrations were quantified by ELISA. The Mann-Whitney U-test was applied for calculation of statistical significance (n.s.= not significant); ΔCAMP = (t = 6) – (t = 0)

	CAMP [ng/mL]	
	rho	*P*
skinfold [mm]	-0.384	<0.001**
excluded confounder		
age [y]	-0.304	0.006**
+ BMI [kg/m²]	-0.396	<0.001**
+ triglycerides [mg/dL]	-0.401	<0.001**
+ total cholesterol [mg/dL]	-0.406	<0.001
+ HDL cholesterol [mg/dL]	-0.283	0.014*
+ LDL cholesterol [mg/dL]	-0.282	0.015*
+ DPP-4 [pg/mL]	-0.275	0.019*

Supplementary Table S2: Significant and positive correlation of CAMP and skinfold persisted after exclusion of confounders

Partial correlation analysis of CAMP and skinfold was performed. Confounders like age, BMI, triglycerides, total cholesterol, HDL cholesterol, LDL cholesterol or DPP-4 can be excluded. (* $p < 0.05$, ** $p < 0.01$).

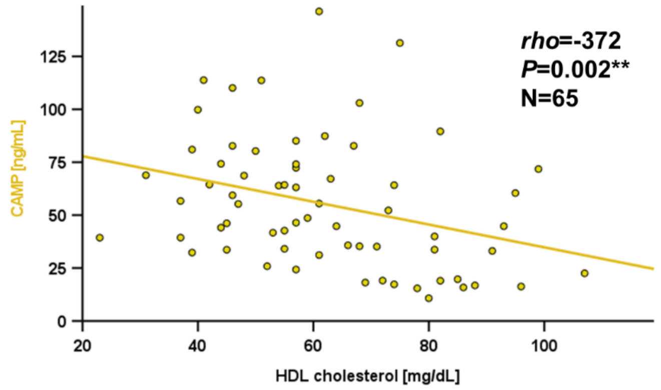

Supplementary Figure S3: HDL cholesterol levels correlated negatively with CAMP serum concentrations in the subgroup of non-smokers

Serum CAMP and HDL cholesterol levels are negatively correlated in the subgroup of non-smokers (n = 65). CAMP serum concentrations were measured by ELISA. Spearman-rho test was applied for calculation of correlation coefficient and statistical significance (** $p < 0.01$).

	CAMP [ng/mL]	
	rho	*P*
HDL cholesterol [mg/dL]	-0.385	<0.001**
excluded confounder		
age [y]	-0.345	0.002**
+ BMI [kg/m²]	-0.355	0.001**
+ triglycerides [mg/dL]	-0.355	0.002**
+ total cholesterol [mg/dL]	-0.423	<0.001**
+ LDL cholesterol [mg/dL]	-0.261	0.024*
+ DPP-4 [pg/mL]	-0.240	0.039*

Supplementary Table S3: Significantly and negatively correlation of CAMP and HDL cholesterol persisted after exclusion of confounders

Partial correlation analysis of CAMP and HDL cholesterol was performed. Confounders like age, BMI, triglycerides, total cholesterol, LDL cholesterol, or DPP-4 can be excluded. (* $p < 0.05$, ** $p < 0.01$).

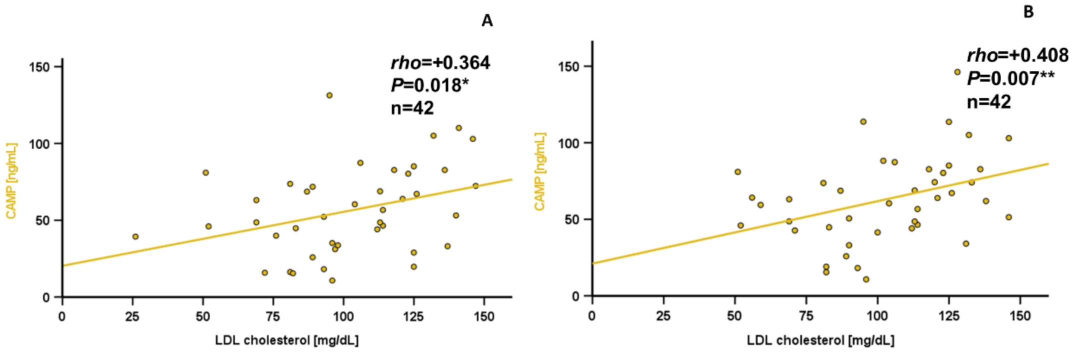

Supplementary Figure S4: LDL cholesterol levels correlated positively with CAMP serum concentrations in the subgroup of patients with low waist-hip-ratio (A) and small skinfold (B)

Serum CAMP and LDL cholesterol levels were positively correlated in the subgroups of patients with low waist-hip-ratio (A) (n = 42) and small skinfold (B) (n = 42). Low waist-hip-ratio was defined as lower than the median of the cohort (WHR < 0.84). Small skinfold was defined as lower than the median of the cohort (skinfold < 10mm).

CAMP serum concentrations were measured by ELISA. Spearman-rho test was applied for calculation of correlation coefficient and statistical significance (* $p < 0.05$,** $p < 0.01$).

	CAMP [ng/mL]	
	rho	*P*
Adiponectin [µg/mL]	-0.224	0.046*
confounder		
age [y]	-0.198	0.080
BMI [kg/m²]	-0.198	0.080

Supplementary Table S4: Partial correlation analyses revealed age or BMI as confounders of significant negative correlation of CAMP and adiponectin

Partial correlation analysis of CAMP and adiponectin was performed. Confounders like age or BMI seem to be relevant confounders of significant negative correlation of CAMP and adiponectin. (* $p < 0.05$)

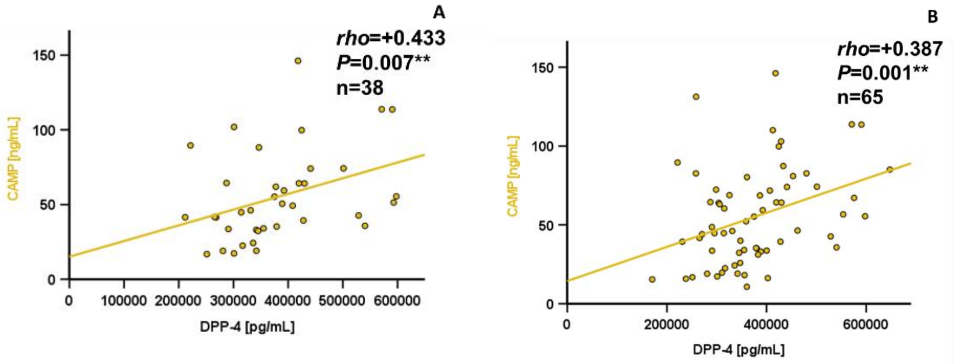

Supplementary Figure S5: DPP-4 levels correlated positively with CAMP serum concentrations in the subgroup of patients with elevated waist-hip-ratio (A) and non-smokers (B)

Serum CAMP and DPP-4 levels are positively correlated in the subgroups of patients with high waist-hip-ratio (A) (n = 38) and non-smokers (B) (n = 65). High waist-hip-ratio was defined as higher than the median of the cohort (WHR > 0.84).

CAMP serum concentrations were measured by ELISA. Spearman-rho test was applied for calculation of correlation coefficient and statistical significance (** $p < 0.01$).

A		
**metabolic parameters (t=0), n=80**	**CAMP [ng/mL] (t=0)**	
	rho	*P*
BMI [kg/m²]	+0.063	n.s.
LDL cholesterol [mg/dL]	+0.188	n.s.
Total cholesterol [mg/dL]	+0.023	n.s.
Triglycerides [mg/dL]	+0.195	n.s.
Glucose [mg/dL]	+0.126	n.s.
Insulin [mU/L]	-0.206	n.s.
**metabolic parameters (t=0), n=80**	**ΔCAMP [ng/mL] (t=(t=6) – (t=0))**	
Skinfold [mm]	+0.127	n.s.
BMI [kg/m²]	-0.133	n.s.
HDL cholesterol [mg/dL]	+0.186	n.s.
LDL cholesterol [mg/dL]	-0.175	n.s.
Total cholesterol [mg/dL]	-0.077	n.s.
Triglycerides [mg/dL]	-0.028	n.s.
Glucose [mg/dL]	-0.008	n.s.
Insulin [mU/L]	+0.170	n.s.
**Δmetabolic parameters (t=(t=6) – (t=0)), n=80**	**ΔCAMP [ng/mL] (t=(t=6) – (t=0))**	
HDL cholesterol [mg/dL]	-0.093	n.s.
LDL cholesterol [mg/dL]	-0.001	n.s.
Total cholesterol [mg/dL]	+0.048	n.s.
Triglycerides [mg/dL]	+0.031	n.s.
Glucose [mg/dL]	-0.059	n.s.
Insulin [mU/L]	-0.042	n.s.
DPP-4 [pg/mL]	+0.009	n.s.
**B**		
**metabolic parameters BMI < 25kg/m² (t=0), n=52**	**CAMP [ng/mL] (t=0)**	
Skinfold [mm]	-0.260	0.062
BMI [kg/m²]	+0.248	n.s.
Total cholesterol [mg/dL]	+0.145	n.s.
Glucose [mg/dL]	+0.024	n.s.
Insulin [mU/L]	-0.099	n.s.
DPP-4 [pg/mL]	+0.254	0.069
**metabolic parameters BMI > 25kg/m² (t=0), n=28**	**CAMP [ng/mL] (t=0)**	
BMI [kg/m²]	-0.252	n.s.
HDL cholesterol [mg/dL]	-0.352	0.066
Total cholesterol [mg/dL]	-0.058	n.s.
Triglycerides [mg/dL]	-0.008	n.s.
Glucose [mg/dL]	+0.213	n.s.

Supplementary Table S5 Correlation analysis of CAMP and metabolic parameters

CAMP serum concentrations were measured by ELISA. Spearman-rho test was applied for calculation of correlation coefficient and statistical significance (* $p < 0.05$). t = hours after oral lipid ingestion. A) total study cohort (n = 80); B) subgroup analyses: BMI < 25kg/m² (n = 52); BMI > 25kg/m² (n = 28); not significant results are shown; significant results are shown in Table 2. (n.s. = not significant)

bile acids (t=0), n=80	CAMP [ng/mL] (t=0)	
	rho	*P*
Total bile acids [nmol/L]	+0.056	n.s.
Free bile acids [nmol/L]	+0.215	0.056
Glycine-conj. bile acids [nmol/L]	+0.028	n.s.
**bile acids (t=0), n=80**	**ΔCAMP [ng/mL] (t=t6-t0)**	
Total bile acids [nmol/L]	+0.044	n.s.
Free bile acids [nmol/L]	-0.147	n.s.
Glycine-conj. bile acids [nmol/L]	+0.058	n.s.
**bile acids (t=0), n=80**	**ΔCAMP [ng/mL] (t=t2-t0)**	
Total bile acids [nmol/L]	+0.218	n.s.
Free bile acids [nmol/L]	-0.046	n.s.
Glycine-conj. bile acids [nmol/L]	+0.186	n.s.
**bile acids, BMI < 25kg/m² (t=0), n=52**	**CAMP [ng/mL] (t=0)**	
Total bile acids [nmol/L]	+0.233	n.s.
Free bile acids [nmol/L]	+0.204	n.s.
Taurine-conj. bile acids [nmol/L]	-0.102	n.s.
Glycine-conj. bile acids [nmol/L]	-0.240	n.s.
**bile acids, BMI > 25kg/m² (t=0), n=28**	**CAMP [ng/mL] (t=0)**	
Total bile acids [nmol/L]	-0.240	n.s.
Free bile acids [nmol/L]	+0.233	n.s.

Supplementary Table S6 Correlation analysis of CAMP and bile acids

CAMP serum concentrations were measured by ELISA. Spearman rho test was applied for calculation of correlation coefficient and statistical significance. (* $p < 0.05$). t = hours after oral lipid ingestion. (significant results are shown in Table 3), n.s. not significant
